# Esophageal pleomorphic rhabdomyosarcoma: a rare high-grade sarcoma managed with upfront resection

**DOI:** 10.1093/jscr/rjaf903

**Published:** 2025-11-13

**Authors:** Jason Stanton, Salmaan Zafer, Jacqueline Lee, Anthony Cipriano

**Affiliations:** Department of Surgery, Geisinger Commonwealth School of Medicine, 525 Pine Street, Scranton, PA 18509, United States; Department of Surgery, Geisinger Commonwealth School of Medicine, 525 Pine Street, Scranton, PA 18509, United States; Department of Thoracic Surgery, Geisinger Wyoming Valley, 1000 East Mountain Boulevard, Wilkes Barre, PA 18711, United States; Department of Thoracic Surgery, Geisinger Community Medical Center, 1800 Mulberry Street, Scranton, PA 18510, United States

**Keywords:** esophageal sarcoma, pleomorphic rhabdomyosarcoma, soft tissue sarcoma, esophagectomy

## Abstract

Primary esophageal sarcomas are exceedingly rare, with a subtype of pleomorphic rhabdomyosarcoma (PRMS) as exceptionally uncommon. We present the case of a healthy 58-year-old male with significant smoking history with 3 months of progressive dysphagia and 30 lb weight loss. Endoscopy revealed a large esophageal mass with biopsy demonstrating a high-grade malignant neoplasm with spindle cell features, favoring sarcoma. Staging workup confirmed a localized intraluminal tumor without distant metastasis. Given the progressive nature of the mass and near-complete obstruction, the patient underwent immediate esophagectomy. Final pathology demonstrated an 8.7 cm high grade PRMS involving the mucosa and submucosa without lymph node involvement, Stage IIIA (pT2N0M0), based on the American Joint Commission on Cancer (AJCC) criteria. This case underscores the importance of distinguishing sarcomas from esophageal carcinomas and recognizing early surgery as the cornerstone of management in localized PRMS given its limited responsiveness to chemoradiation.

## Introduction

Primary esophageal malignancies are most commonly squamous cell carcinomas and adenocarcinoma, which together account for more than 95% of cases worldwide [1]. Sarcomas of the esophagus are remarkably rare, representing <1% of esophageal tumors [2]. Among them, pleomorphic rhabdomyosarcoma (PRMS), an aggressive subtype of rhabdomyosarcoma usually seen in adults, is exceedingly uncommon when arising from the esophagus [3].

Rhabdomyosarcomas are soft tissue sarcomas of skeletal muscle origin, more often encountered in pediatric populations. PRMS in adults is characterized by high-grade morphology, aggressive clinical course, and poor response to conventional chemoradiation [4]. The natural progression of disease differs from that of esophageal carcinoma; sarcomas tend to spread predominantly via hematogenous dissemination rather than lymphatic, and nodal involvement is therefore relatively uncommon [5].

Primary esophageal PRMS is an uncommon subtype, with <20 cases reported in the literature, highlighting the rarity and challenges that these tumors present [6]. Given the limited published data, there is no standardized management of esophageal PRMS. Most reports emphasize surgical resection as the basis of treatment [2, 7]. Furthermore, the American Joint Commission on Cancer (AJCC) recommends staging esophageal sarcoma according to the visceral soft tissue sarcoma criteria, with an emphasis on tumor size and grade rather than depth of invasion or nodal status [8].

## Case report

**Figure 1:**
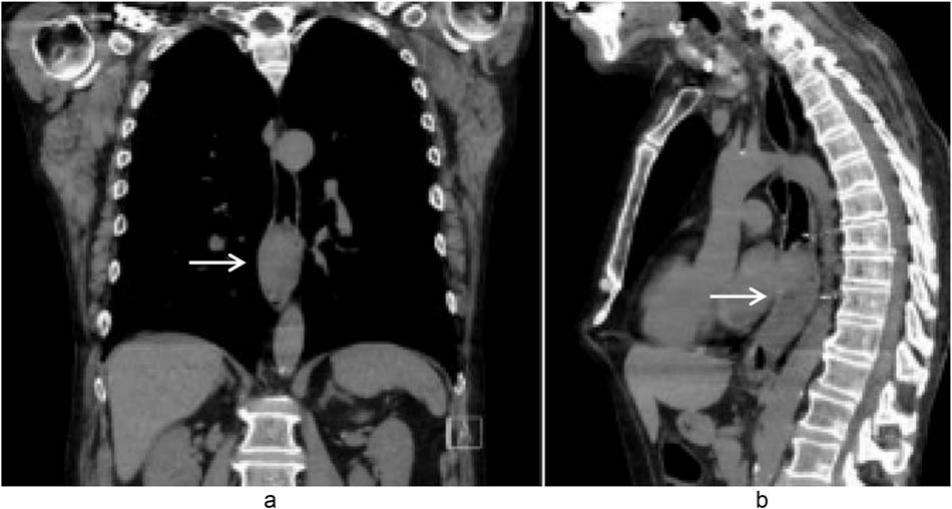
Coronal (a) and sagittal (b) views of the initial CT scan at time of presentation that revealed a large esophageal mass with concern for complete obstruction (arrows).

**Figure 2:**
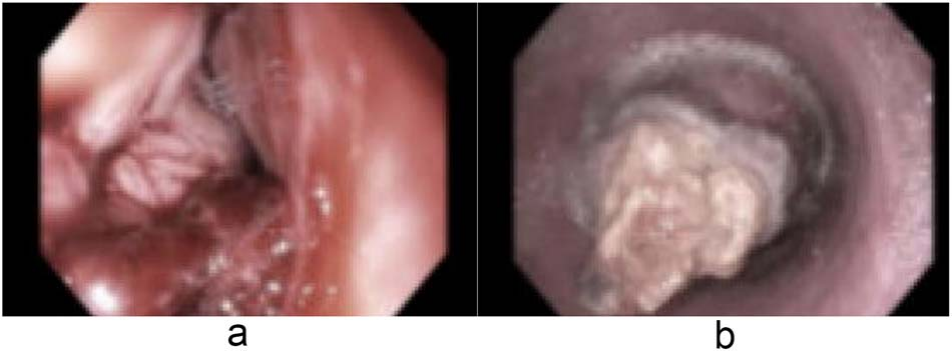
Endoscopic images (a and b) of a nearly obstructive intraluminal esophageal mass.

**Figure 3:**
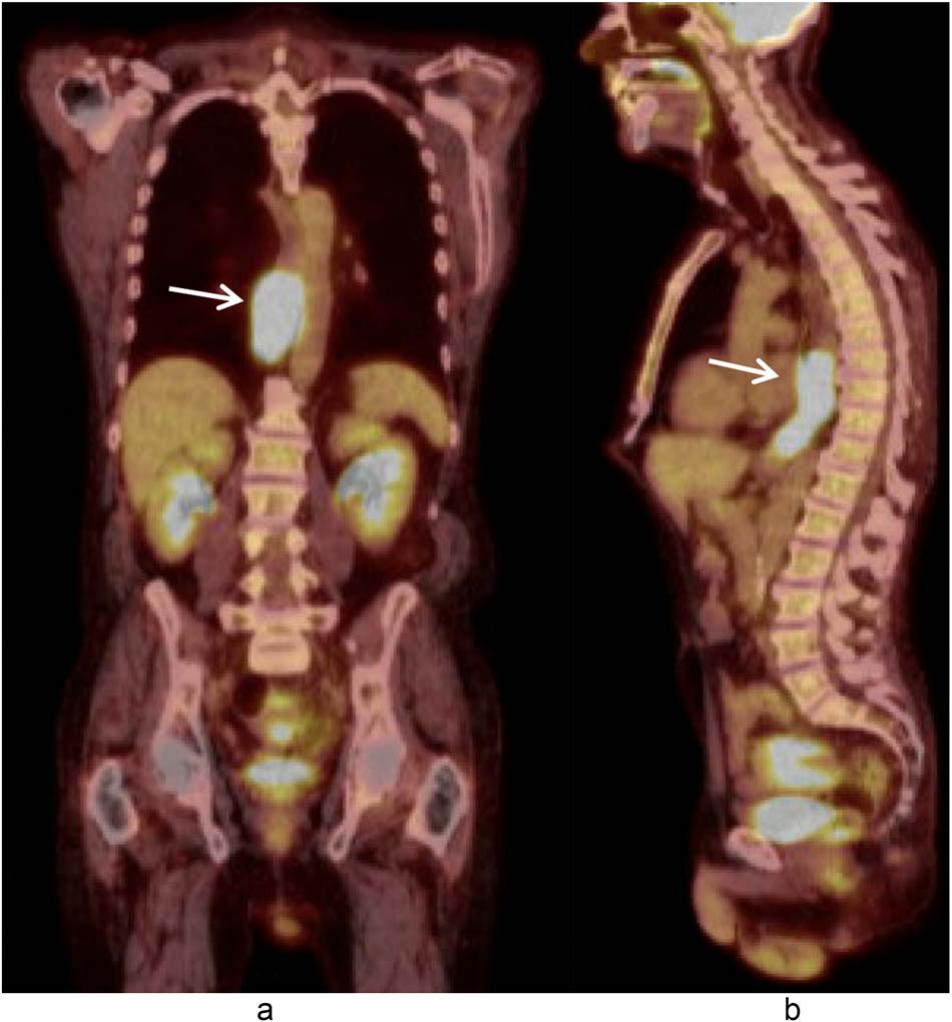
Coronal (a) and sagittal (b) views of the PET scan showing area of hypermetabolic activity at the lower esophagus without evidence of distant disease (arrows).

A 58-year-old male with a history of hypertension, daily alcohol use, and 35 pack year smoking history presented with progressive dysphagia and significant weight loss over a period of 3 months. His symptoms began with heartburn, with initial failure of empiric treatment for reflux. He then developed dysphagia to solids that progressed to liquids and eventually an inability to tolerate his own saliva. Over that timeframe, he also noted unintentional weight loss of ~30 lbs. He eventually presented to the emergency department where a CT scan raised concern for an obstructing esophageal mass (Fig. 1). He urgently underwent upper endoscopy revealing a large polypoid tumor with a broad base located in the lower esophagus (Fig. 2). Biopsies revealed a high-grade malignant neoplasm with spindle cell features concerning for a sarcoma. Further workup with brain MRI and positron emission tomography (PET) scan confirmed a hypermetabolic esophageal mass without evidence of distant metastatic disease (Fig. 3). Unfortunately, endoscopic ultrasound was unable to be performed given the nearly obstructive nature of the tumor.

**Figure 4:**
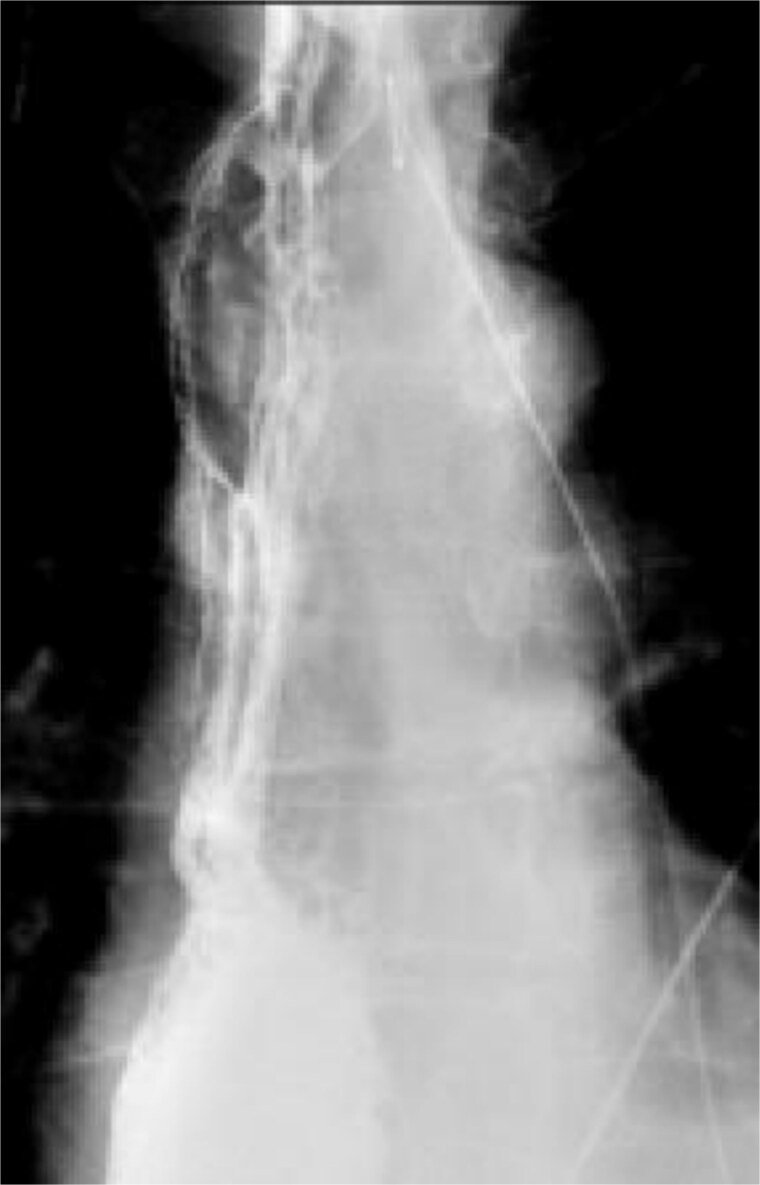
Fluoroscopic esophagram showing an intact anastomosis without contrast extravasation on postoperative day 6 status post-esophagectomy.

**Figure 5:**
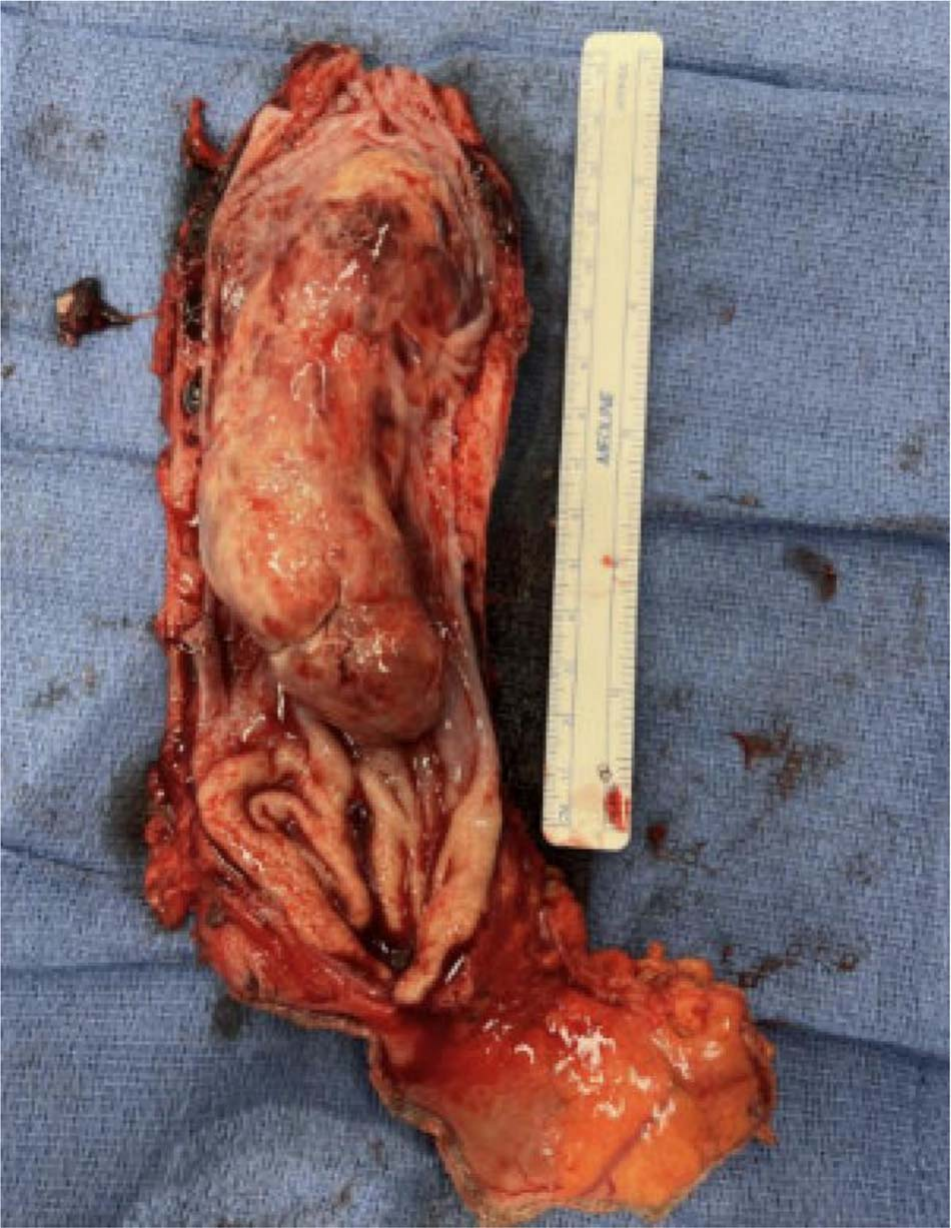
Gross specimen of esophageal mass.

A multidisciplinary discussion was held and in the setting of the rapid tumor progression with biology favoring sarcoma and no evidence of distant disease, the decision was made to pursue upfront surgical resection rather than neoadjuvant therapy. The patient underwent a three field near total esophagectomy with an uneventful postoperative hospital course. A fluoroscopic esophagram was performed on postoperative day 6 without evidence of contrast extravasation (Fig. 4). The final pathology revealed an 8.7 cm high-grade pleomorphic rhabdomyosarcoma with mucosal and submucosal invasion, sparing the muscularis propria with 0/11 lymph node involvement (Fig. 5). This was formally graded as Stage IIIA (pT2N0M0), based on the soft tissue sarcoma criteria of AJCC.

## Discussion

Esophageal PRMS represents an exceptionally rare malignancy, with only a handful of cases reported in the literature. This case represented the typical clinical presentation of this unusual pathology; with rapidly progressive dysphagia, significant weight loss, and eventual obstructing intraluminal mass, as seen in prior descriptions of primary esophageal sarcoma [2, 6]. Unlike squamous cell carcinoma and adenocarcinoma of the esophagus, sarcomas follow a distinct but different biologic course. They will typically spread hematogenously, most often to the liver or lungs, while nodal involvement is uncommon [5, 6]. This difference in metastatic pattern explains why, in this case, lymph nodes were uninvolved despite large tumor size and high-grade features.

Staging of esophageal sarcomas also requires a different framework when compared to squamous cell or adenocarcinoma. While esophageal carcinomas are staged according to depth of invasion and nodal involvement, the AJCC recommends that esophageal sarcomas be staging prioritizing tumor size and histologic grade [8]. In our patient, despite lack of muscularis propria invasion and absence of nodal disease, the large tumor size and high-grade histology warranted classification as Stage IIIA (pT2N0M0). This again highlights the prognostic importance of size and grade in sarcoma cancer biology, as opposed to depth-based staging in carcinoma.

The therapeutic approach likewise differs substantially. With esophageal carcinoma, multimodality therapy with neoadjuvant chemoradiation followed by surgery is standard for locally advanced disease. In contrast, for esophageal sarcomas, surgical resection remains the primary treatment modality. Adult PRMS demonstrated limited responsiveness to chemotherapy or radiation [4, 9]. For this patient, upfront surgical resection was favored not only to relieve his near-complete obstruction, but also to achieve definitive diagnosis and accurate staging. This multidisciplinary decision aligns with the current literature, emphasizing the importance of early resection for locally aggressive sarcomas in order to optimize outcomes [2, 7].

This case highlights the unique clinical, pathological, and therapeutic challenges faced when managing a patient with esophageal pleomorphic rhabdomyosarcoma. The patient’s presentation with severe and rapidly progressing dysphagia and significant weight loss underscored the aggressive nature of this disease, while final pathology confirmed its rarity and high-grade features. Unlike esophageal carcinoma, in which depth of invasion and nodal status are central to staging and prognosis, esophageal sarcomas are best categorized using tumor size and histologic subtype. For this patient, these factors placed him at Stage IIIA despite absence of muscularis propria invasion.

Management decisions must also account for the poor responsiveness of adult PRMS to conventional chemoradiation. In this context, upfront surgical resection remains the most effective strategy, offering both symptomatic relief and definitive staging. This case reinforces the importance of accurate histopathologic diagnosis and thoughtful application of sarcoma staging guideless to guide therapy in the setting of a relative paucity of literature guiding appropriate management. Continued reporting of such rare cases will be essential to improving collective understanding and developing an evidence-based management strategy for esophageal sarcoma.
